# Draft Genome Sequence of the Opportunistic Human Pathogen *Morganella morganii* SC01

**DOI:** 10.1128/genomeA.00051-12

**Published:** 2013-01-24

**Authors:** Indu Khatri, Chetna Dureja, Saumya Raychaudhuri, Srikrishna Subramanian

**Affiliations:** CSIR-Institute of Microbial Technology, Chandigarh, India

## Abstract

We report the 4.1-Mb draft genome sequence of *Morganella morganii* SC01, a gammaproteobacterium, isolated from an Indian human fecal sample.

## GENOME ANNOUNCEMENT

*Morganella morganii*, a member of the tribe *Proteae* of the family *Enterobacteriaceae*, is a Gram-negative bacillus bacterium. It is a facultative anaerobe often regarded as a harmless opportunistic pathogen. However, some strains may carry transmissible antibiotic-resistance-conferring plasmids and can cause large nosocomial outbreaks of infection with serious morbidity and mortality (1).

The genome of *M. morganii* SC01 was purified from human feces in accordance with the guidelines of our institutional bioethics committee (clearance number IBSC/2012-2/19) and sequenced using the Illumina-HiSeq 1000 platform. Sequencing resulted in 12,194,768 paired-end reads (insert size of 350 bp) of length 101 bp. A total of 12,083,811 high-quality reads with approximately 295× coverage were extracted and assembled using CLCbio wb5.5 (http://www.clcbio.com) (word size 45 and bubble size 60) to obtain a 4.1-Mb genome with 90 contigs (N_50_, 215,046 bp). Functional annotation was carried out using RAST (Rapid Annotation using Subsystem Technology) (2), tRNA was predicted by tRNAscan-SE-1.23 (3), and rRNA genes were predicted by RNAmmer 1.2 (4). The genome contains three rRNA genes (5S-23S-16S) and 74 aminoacyl-tRNA synthetase genes. A total of 4,099 coding regions were predicted in the genome, of which 3,025 (74%) could be functionally annotated. The genome coding density is 86.9% with an average gene length of 865 bp. The annotated genome has 73 genes involved in virulence, disease, and defense, including 52 genes for resistance to antibiotics and toxic compounds. Three genes are involved in dormancy and sporulation of the bacterium. The genome also contains 20 genes for sulfur metabolism and 30 genes for phosphorus metabolism. The functional comparisons of genome sequences available on the RAST server revealed the closest neighbors of *M. morganii* SC01 as *Providencia rustigianii* DSM4541 (score 538) followed by *Providencia stuartii* ATCC 25827 (score 295), *Providencia rettgeri* DSM 1131 (score 278), and *Proteus mirabilis* ATCC 29906 (score 270).

### Nucleotide sequence accession numbers.

This Whole Genome Shotgun project has been deposited at DDBJ/EMBL/GenBank under the accession number AMWL00000000. The version described in this paper is the first version, AMWL01000000.
